# Temperature Uncertainty Analysis of Injection Mechanism Based on Kriging Modeling

**DOI:** 10.3390/ma10111319

**Published:** 2017-11-17

**Authors:** Dongdong You, Dehui Liu, Xiaomo Jiang, Xueyu Cheng, Xiang Wang

**Affiliations:** 1 National Engineering Research Center of Near-Net-Shape Forming for Metallic Materials, South China University of Technology, Guangzhou 510640, China; liudehuimg@foxmail.com (D.L.); wong_cheung@foxmail.com (X.W.); 2 College of Engineering, Tongji University, Shanghai 200092, China; 3 Applied Science, Clayton State University, Morrow, GA 30260, USA; XueyuCheng@clayton.edu

**Keywords:** uncertainty, injection mechanism, squeeze casting, kriging modeling, numerical simulation, friction

## Abstract

A kriging modeling method is proposed to conduct the temperature uncertainty analysis of an injection mechanism in squeeze casting. A mathematical model of temperature prediction with multi input and single output is employed to estimate the temperature spatiotemporal distributions of the injection mechanism. The kriging model applies different weights to the independent variables according to spatial location of sample points and their correlation, thus reducing the estimation variance. The predicted value of the kriging model is compared with the sample data at the corresponding position to investigate the influence of the temperature uncertainty of the injection mechanism on the injection process including friction. The results indicate that the significant error is observed at a few sample points in the early injection due to the impact of the uncertainty facts. The variance mean and standard deviation obtained by the model calibrated by experimental samples reduce largely in comparison to those obtained from the initial kriging model. This study indicates that model calibration produces more accurate prediction.

## 1. Introduction

In squeeze casting, the temperature and deformation of injection mechanism are two significant parameters of the injection process. Numerical simulation methods have been widely applied in the research and a state-of-the-art literature review of relative research has been conducted in our previous work [1]. In these studies, however, it is observed that the model and boundary conditions are simplified based on the assumption of deterministic parameters under ideal conditions. Many uncertainty factors impact the injection process, such as temperature non-uniformity of shot sleeve, punch and metal melt; structural asymmetry of shot sleeve; manufacturing and assembling error of shot sleeve and punch; non-uniformity of friction coefficient and heat transfer coefficient, and testing data error. The error between numerical and actual results may largely impact the prediction accuracy of numerical models. Quantitative assessment of uncertainty factors and model modification under uncertainties have become two significant issues in the optimal design and reliability analysis of squeeze casting using numerical simulation or experimental methods.

Uncertainty analysis methods have been broadly employed in different applications such as numerical data optimization, product lifetime prediction, and mechanical optimization design [2,3,4,5,6]. The uncertainty is usually classified into aleatoric and epistemic ones. The former is irreducible, as in inherent variability, such as statistically distributed properties and manufacturing variability, while the latter is potentially reducible uncertainty due to lack of knowledge, such as model form and initial and boundary condition approximations. Recently, uncertainty modeling and optimization of the casting process has also attracted a considerable number of researchers (e.g., [7,8,9,10,11,12,13,14]). Tao et al. [7] proposed a reliability-based multidisciplinary optimization model through finite element analysis of the die-casting process, in which the evidence theory is used to represent the epistemic uncertainty. Hardin et al. [8] conducted casting simulation with a reliability based design optimization (RBDO) software that considers uncertainties in both the input variables as well as in modeling of itself. The riser design in the casting process example was optimized. Fezi et al. [9] performed uncertainty quantification and sensitivity analysis on a transient model of transport phenomena during the solidification of grain refined Al-Cu alloy, and investigated the effect of various uncertainties in microstructural model parameters, thermal boundary conditions and material property inputs on macrosegregation levels and solidification time. Plotkowski et al. [10] employed the PRISM uncertainty quantification framework to investigate the effect of input uncertainty on the output of grain attachment models for the equiaxed solidification of multicomponent alloys. Wang et al. [11] improved the measurement accuracy of heat transfer coefficients under uncertainty and large disturbance by using an integrated approach to predict the solidified shell thickness of billets. Wu et al. [12] addressed uncertainties due to model parameters and assumptions in multiphase solidification simulations including a unidirectional solidification and a cylindrical ingot casting. Carlson et al. [13] addressed uncertainties in the casting simulation through developing an iterative method to promote the material parameter accuracy. Sakalli et al. [14] handled the aleatory and epistemic uncertainties simultaneously in a blending optimization problem for brass casting and transformed the possibilistic uncertainties into probabilistic ones. Clearly, to reduce errors while improving model reliability and accuracy of the injection mechanism, the uncertainty of various parameters and data should be modeled and quantitatively analyzed by introducing the uncertainty analysis method. In addition, it is necessary to quantitatively evaluate the model and calibrate the model parameters with experimental results so as to control the uncertainty and improve the injection mechanism design.

Recently, as the commonly-used metamodeling technique, the kriging model has been widely applied in the fields of simulation validation, reliability evaluation, and optimal design [15,16,17,18,19,20,21,22,23]. The kriging model is an optimal Gauss interpolation process based on regression analysis of simulation or experimental data and weighted according to spatial covariance values. It outperforms other surrogate modeling techniques due to its unique characteristics of spatiotemporal modeling. In particular, regarding materials forming, a lot of research based on kriging model technique has been conducted (e.g., [24,25,26,27,28,29,30,31,32,33,34,35,36,37,38,39]). Tuo et al. [24] proposed a kriging model based on a nonstationary Gaussian process that integrates the outputs of different mesh densities in finite element analysis and provides approximation to the exact solution, and then applied the model in casting simulation. Hofwing et al. [25] performed the robustness analysis of residual stresses in the brake discs casting by Monte Carlo simulations of metamodels, considering quadratic response surfaces and kriging approximations. Li et al. [26] presented a hybrid inverse identification approach by combining the finite element method, kriging model, Latin hypercube sampling and multi-island genetic algorithm to deal with material parameters of aluminum alloy. Deng et al. [27] presented a kriging surrogate modeling strategy to substitute the computationally intensive numerical simulation of injection molding process, with the purpose of obtaining the optimal process parameters. Li et al. [28] proposed a hybrid method to optimize the stent microinjection molding process by combining the finite element analysis with the kriging modeling technique. Wang et al. [29] presented a kriging model-based sequential optimization method to reduce the warpage of three kinds of products in plastic injection molding. Kang et al. [30] adopted a metamodel-based design optimization approach to optimally determine the design variables of injection molding process, using kriging models to replace time-consuming numerical simulations. Dang et al. [31] compared several commonly-used optimization approaches, such as kriging model, response surface model, artificial neural network, etc. and proposed a meta-modeling optimization framework for simulation-based optimization of injection molding process parameters. Gao et al. [32] developed the stepwise searching method based on kriging metamodels of defect indexes to determine the forming limit, considering defects in the transitional region during local loading forming of Ti-alloy large-scale rib-web components. Ambrogio et al. [33] designed a metamodelling technique by integrating the design of the experimental statistical method and the kriging one and validated the feasibility of the proposed method for the crucial problem of localized thinning in the sheet metal forming process. Abebe et al. [34] applied an ordinary kriging model-based prediction technique to find the wrinkling and dimple occurrence limit on metallic alloys multi-point dieless forming. Tutum et al. [35] employed the kriging surrogate method for thermochemical simulation of the pultrusion process. Kusiak et al. [36] compared the effectiveness of three metamodelling techniques: kriging method, response surface methodology and artificial neural network, in the optimization of laminar cooling of rolled Dual Phase (DP) steel strips. Meng et al. [37] presented a kriging based multi-objective optimization methodology for the theoretic pareto optimal front in the forging process problem. A two-step forging problem of an aeronautic component is employed to show the efficiency of the proposed methodology. Roux et al. [38] dealt with the optimization of the clinching process using a global optimization technique based on the kriging meta-model. Its purpose is to optimize the mechanical strength for tensile loading and shear loading of the clinched component. An et al. [39] used the kriging model to optimize the loading parameters in constraint handling of a pre-bending process prior to hydroforming.

In this paper, on the basis of the numerical and experimental results in the previous work [1], the kriging modeling methodology is developed to establish the uncertainty analysis model of injection mechanism in squeeze casting. According to the spatiotemporal distribution characteristics of temperature variable in the injection mechanism, a multiple-inputs-single-output (MISO) predictive mathematical model is established. Different weights are assigned to independent variables by making full use of the spatial location of sample points and their correlation to minimize estimation variance. The comparison study between the model prediction and sample data indicates that the kriging modeling method is feasible in uncertainty analysis of injection mechanism.

## 2. Kriging Modeling Methodology of Injection Mechanism

### 2.1. Meta-Modeling Method

The accurate mathematical model y=f(x) is often difficult to obtain for a complex system. An approximate surrogate model is usually established from the fitting algorithm [40] by using the input/output sample data $\left\{x^{i}\right\}$ and $\left\{y^{i}\right\}$ obtained from the test or simulation method, given as follows:

(1) $$\hat{y}=g(x),$$

where $\hat{y}$ is the output of the meta-model. The relationship between $\hat{y}$ and the actual output y is:

(2) $$y=\hat{y}+\varepsilon ,$$

where ε represents the approximate and random errors in the meta-modeling.

The kriging method is adopted to establish the uncertainty meta-model of injection mechanism. The objectives are as follows:

1. Avoid time-consuming simulation and reduce the iteration time;
2. Filtering out the possible numerical noise produced by the original analysis model;
3. Estimate the response relationship between input and output parameters;
4. Avoid the local optimal solution effectively; find the global solution using numerical algorithm and shorten the optimization period;
5. Form better optimization strategy with other algorithms, such as Design of Experiments (DOE), Optimization, Robust Design and so on.

### 2.2. Uncertainty Problem Description of Injection Mechanism Based on Meta-Model

**Figure 1:**
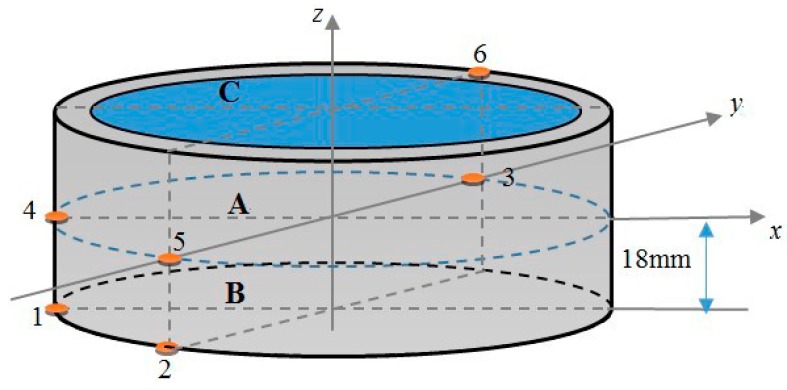
Thermocouples placement in 3D Cartesian coordinates.

The spatiotemporal predictive model is MISO, i.e., given coordinates (x,y,z), time (t) and friction condition $\left(I_{f}\right)$, the model predicts the temperature T at different locations of the shot sleeve and the punch under uncertain conditions. The mathematical expression is given as follows:

(3) $$T=f(x,y,z,t,I_{f},I_{s}),$$

where $I_{f}$ is the friction condition index, indicating friction (=1) or not (=0); $I_{s}$ is the position index, indicating the punch (=1) or the shot sleeve (=2). The coordinates of the sample points are shown in Figure 1, which shows the thermocouples 1–6 installed on the inner wall of the shot sleeve. Section A, which has the same altitude as the top surface of the punch, is also the lowest surface of the melt. Sections B and C are below and above a proper distance of section A. Refer to You et al. [1] for details about the squeeze casting process and the experimental measurement.

In this study, 1219 original sample points are collected from the experimental and numerical data. The sample data points are divided into two parts: training and testing samples for model building and testing, respectively. Considering the integrity of the data in modeling, the data segmentation is randomly selected from six locations. In addition, 548 simulation data are collected from the six positions shown in Figure 1, among which 478 sample data are utilized for kriging meta-modeling, and the others are used for model testing. Furthermore, 553 experimental data points are used to calibrate the kriging model, and the other 118 sample data points are used to quantitatively validate the calibration model.

### 2.3. Kriging Meta-Modeling of the Injection Mechanism

#### 2.3.1. Mathematical Model

**Figure 2:**
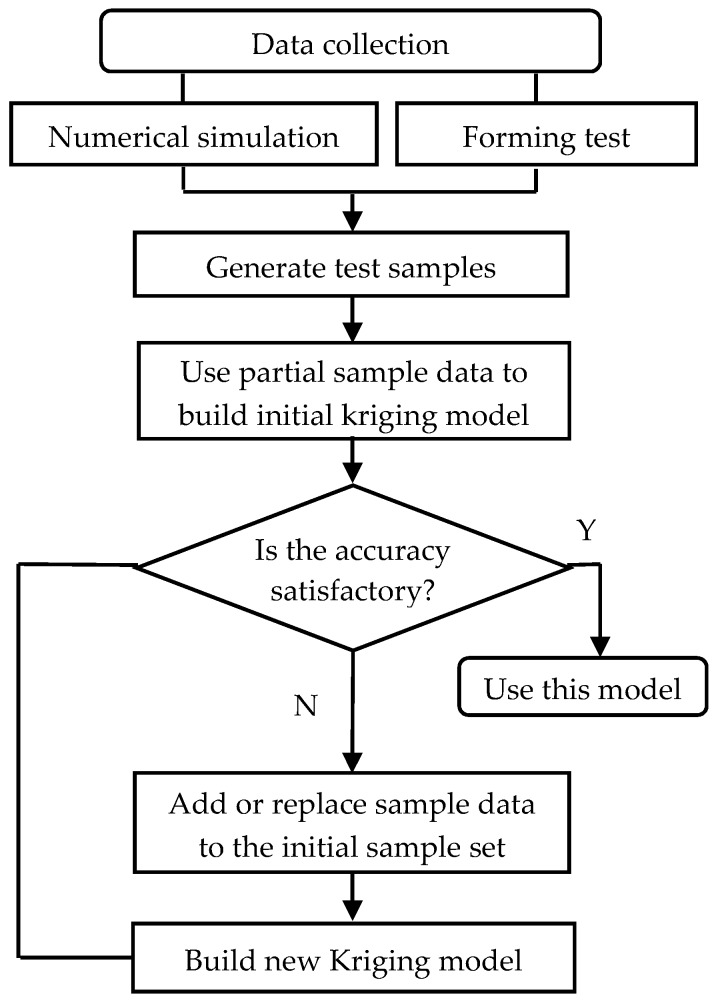
Implementation process of kriging meta-modeling.

In this study, the squeeze casting machine is regarded as a system and each component such as punch, shot sleeve, etc. is considered as a subsystem. The kriging modeling includes the following three steps:

1. According to the test requirements, determine the position of the sample point $x_{i}\;(i=1,2,\ldots ,n)$, where $x_{i}=[x_{1},x_{2},\ldots ,x_{m}]$ is an *m*-dimensional point;
2. Obtain the response value $y_{i}$ at sample point $x_{i}$ by numerical simulation or experiment to form complete sample data $\left\{(x_{i},y_{i}),i=1,2,\ldots ,n\right\}$, where $y_{i}=[y_{1},y_{2},\ldots ,y_{q}]$ is a vector representing the *q*-dimensional response values;
3. Using partial sample data, build the appropriate kriging model f(x) to make $f(x_{i})$ and $y_{i}$ fit well. Then, check and calibrate the model with the other sample data. Thereafter, iterative computations are performed until the calibration model meets the precision requirements. The flow chat is shown in Figure 2.

By Equation (3), each sample point has six independent variables, so $x_{i}=[x_{1},x_{2},\ldots ,x_{m}]$ is a six-dimensional space sample point, namely, *m* = 6. The response value $y_{i}=[y_{1},y_{2},\ldots ,y_{q}]$ is one-dimension, i.e., *q* = 1. Moreover, *n* = 1219 complete sample data $\left\{(x_{i},y_{i}),i=1,2,\ldots ,n\right\}$ are collected.

The kriging model can be defined as the combination of a regression model and a stochastic process:

(4) y(x)=F(β,x)+z(x),

where $F(\beta ,x)=f{\left(x\right)}^{T}\beta =\sum_{k=1}^{p}\beta_{k}f_{k}(x)$ is a regression model of providing the global approximation in the design space, in which $f(x)=[f_{1}(x),f_{2}(x),\ldots ,f_{p}(x){]}^{T}$ is a polynomial of x, with the regression coefficients $\beta ={\left[\beta_{1},\beta_{2},\ldots ,\beta_{p}\right]}^{T}$. The function z(x) provides the approximation of local deviation for the random function, which is assumed to follow a normal distribution with the mean of 0 and the variance of $\sigma_{z}^{2}$. Its covariance is expressed as:

(5) $$\mathrm{cov}[z(x_{i}),z(x_{j})]=\sigma_{z}^{2}R([R(\theta ,x_{i},x_{j})]),$$

where $R(\theta ,x_{i},x_{j})$ is the correlation function with the parameter θ for any two sample data points $x_{i}$ and $x_{j}$. It represents the spatial correlation of the sample points $x_{i}$ and $x_{j}$ and plays an important role in the model accuracy.

The kriging method makes full use of the spatial location of the sample points and their correlation, applying different weights to the response values of each sample so as to minimize the estimated variance [18]. Based on the assumption of model (4), a linearly weighted combination of the response values $Y=\left[y_{1},y_{2},\ldots ,y_{n}\right]$ of the known sample point $S=\left[x_{1},x_{2},\ldots ,x_{n}\right]$ is used as the response estimate of any test point $x_{new}$.

(6) $$\hat{y}(x_{new})=c^{T}Y,$$

where $c={\left[c_{1},c_{2},\ldots ,c_{n}\right]}^{T}$ is the weight coefficient vector to be determined.

The prediction error of the model is

(7) $$\begin{array}{ll} \hat{y}(x_{new})-y(x_{new}) & =c^{T}Y-y(x_{new})\\ & =c^{T}Z-z+{\left(F^{T}c-f\right)}^{T}\beta \end{array},$$

where $F=[f_{1},f_{2},\ldots ,f_{n}]$ and $y(x_{new})$ are the true value; $Z=[z_{1},z_{2},\ldots ,z_{n}{]}^{T}$ is the error of the known sample points.

In order to ensure the unbiasedness of the simulation, assuming that Equation (7) is equal to zero, we obtain:

(8) $$F^{T}c-f=0.$$

Thus, the predictive variance of the Equation (6) is the mean square error (MSE) expressed by:

(9) $$\begin{array}{ll} \sigma^{2}(x_{new}) & =E[{\left(\hat{y}(x_{new})-y(x_{new})\right)}^{2}]\\ & =E[{\left(c^{T}Z-z\right)}^{2}]\\ & =E[z^{2}+c^{T}ZZc-2c^{T}Zz]\\ & =\sigma_{z}^{2}(1+c^{T}Rc-2c^{T}r) \end{array},$$

where $r(x)={\left[R(\theta ,x_{new},x_{1}),R(\theta ,x_{new},x_{2}),\ldots ,R(\theta ,x_{new},x_{n})\right]}^{T}$ indicates the correlation between the test points $x_{new}$ and the sample points S.

For the requirement of minimal variance, a Lagrangian multiplier is introduced to solve the weight coefficient c in Equation (6):

(10) $$\begin{array}{l} L(c,\lambda )=\sigma_{z}^{2}(1+c^{T}Rc-2c^{T}r)-\lambda^{T}(F^{T}c-f)\\ {L^{\prime }}_{c}(c,\lambda )=2\sigma_{z}^{2}(Rc-r)-F\lambda \end{array}.$$

Assuming ${L^{\prime }}_{c}(c,\lambda )=0$, we have:

(11) $$\left\{ \begin{array}{l} \tilde{\lambda }=-\frac{\lambda }{2\sigma_{z}^{2}}={\left(F^{T}R^{-1}F\right)}^{-1}(F^{T}R^{-1}r-f)\\ c=R^{-1}(r-F\tilde{\lambda }) \end{array}\right..$$

Substituting Equation (11) into Equations (6) and (9), respectively, the prediction value and its variance of the test point $x_{new}$ can be obtained as:

(12) $$\hat{y}(x_{new})=f{\left(x_{new}\right)}^{T}\beta^{*}+r{\left(x_{new}\right)}^{T}\gamma^{*},$$

where $\beta^{*}={\left(F^{T}R^{-1}F\right)}^{-1}F^{T}R^{-1}Y$ is solved by the generalized least squares estimation of the regression problem $F\beta^{*}≅Y$; $\gamma^{*}$ is solved by the margin expression $R\gamma^{*}=Y-F\beta^{*}$:

(13) $$\sigma^{2}(x_{new})=\sigma_{z}^{2}(1+u^{T}{\left(F^{T}R^{-1}F\right)}^{-1}u-r^{T}R^{-1}r),$$

where $u=F^{T}R^{-1}r-f$; $\sigma_{z}^{2}={\left(Y-F\beta^{*}\right)}^{T}R^{-1}(Y-F\beta^{*})/n$ represents the variance of the corresponding components of the multidimensional output response.

Equations (12) and (13) constitute the kriging model based on the sample data points (S,Y), which can be used to calculate the predicted value and its variance at any point. For a given sample space, after determining the regression model and the correlation model, the correlation matrix **R** and the derived $\beta^{*}$ and $\sigma_{z}^{2}$ are all dependent on parameter θ, and different θ values will generate different kriging models. The optimal θ value is determined by the method of defining the likelihood function. The optimal kriging model is obtained by solving the unconstrained nonlinear optimization problem of the Equation (14):

(14) $$\frac{n\ln({\hat{\sigma }}_{z}^{2})+\ln\left|R\right|}{2}.$$

#### 2.3.2. Selection of Regression Function

Polynomial regression model intends to simulate the global approximation in the design space, where the polynomial generally consists of the constant, linear and quadratic polynomial items. In the injection process, because of the high nonlinearity of the uncertain temperature variation of the components, the quadratic polynomial is used as the regression model:

(15) $$\begin{array}{l} p=\frac{1}{2}(n+1)(n+2)\\ f_{1}(x)=1,f_{2}(x)=x_{1},\ldots ,f_{n+1}(x)=x_{n}\\ f_{n+2}(x)=x_{1}^{2},\ldots ,f_{2n+1}(x)=x_{1}x_{n}\\ f_{2n+2}(x)=x_{2}^{2},f_{3n}(x)=x_{2}x_{n}\ldots ,f_{p}(x)=x_{n}^{2} \end{array}.$$

#### 2.3.3. Selection of Correlation Function *R*

In the kriging surrogate model, the random distribution function provides the simulated approximation of the local error. The errors that follow the random distribution are not independent but correlated spatially. The correlation between two sample data is related to the distance between the two points, thus yielding the following relation:

(16) $$R(\theta ,x_{i},x_{j})=\prod_{k=1}^{m}R_{k}(\theta_{k},d_{k})\begin{array}{cc} , & d_{k} \end{array}=|x_{i}^{k},x_{j}^{k}|,$$

where *m* is the dimension of design variables; $x_{i}^{k}$ and $x_{j}^{k}$ represent the *k*-dimensional components in the sample vectors $x_{i}$ and $x_{j}$, respectively; $\theta_{k}$ is the regression coefficient of the *k* dimensional component; and the function $R_{k}(\theta_{k},d_{k})$ has various forms, whose kernel functions include exponential function, Gauss function, linear function, spherical function, three-order function, spline function and so on.

The Gauss function $\exp(-\theta_{k}d_{k}^{2})$ is a parabolic one commonly used in continuous differentiable object due to its high computational efficiency, thus chosen as the correlation function in this study. Then, Equation (16) can be expressed as:

(17) $$R(\theta ,x_{i},x_{j})=\exp\left[-{\sum_{k=1}^{m}\theta_{k}\left(x_{i}^{k}-x_{j}^{k}\right)}^{2}\right].$$

Additionally, Equation (14) can be equivalent to a minimization problem:

(18) $$\min_{\theta >0}\{|R{|}^{\frac{1}{n}}\sigma_{z}^{2}\}.$$

**Figure 3:**
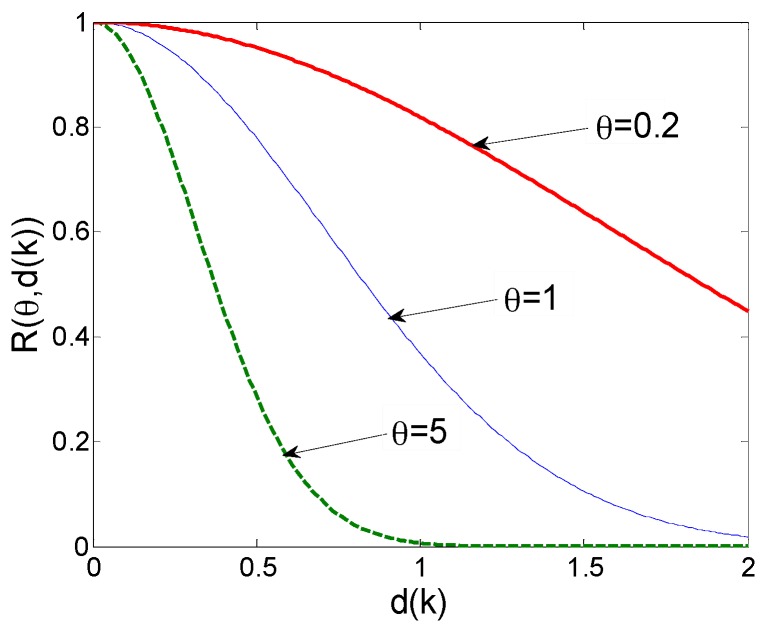
Curves of the Gaussian correlation function.

From the Gaussian function curves shown in Figure 3, the correlation between sample points shows a downward trend with the increase of distance. It is observed that the larger the θ value is, the smaller the correlation area, namely, the faster the correlation declines. For the smaller θ value, the correlation between two points is greater when the distance $\left(d_{k}\right)$ increases, which means that the response values of two points farther apart show an insignificant difference. Therefore, θ is generally regarded as a measure of the importance of independent variables, whose selection directly affects the accuracy of the model.

For the multidimensional model, the dimension of θ should be the same as the number of independent variables, i.e., the dimension of x. The parameter θ values may be the same or different in all directions, corresponding to isotropic or anisotropic problems, respectively. Parameter $\theta_{k}$ represents the weight of the corresponding variable $x_{k}$, which means the influence of $x_{k}$ on its response value. Hence, the importance of variables may be determined by θ. It is observed that the effects of the six independent variables on the temperature response are varying, implying that the θ value is anisotropic in this study.

According to the spatiotemporal temperature distribution of the injection mechanism in the injection process, we make full use of the space location of sample points and their correlation and apply different weights to the sample response values in the establishment of the kriging model. The establishment and solution of the kriging model is programmed in Matlab toolbox DACE (R2014a, The MathWorks, Inc., Natick, MA, USA).

## 3. Results Analysis and Discussion

Two assumptions are made in this study, as follows:

1. Both experiments and simulations are carried out accurately. The main difference between them results from the inaccuracy of material properties, the load transformation and the errors of the numerical model;
2. Data reconstruction is performed on the error points at the six measurement positions. The kriging model is fitted by using the existing points, and then calibrated with the error points.

### 3.1. Kriging Model Related Parameters

According to the procedure of Figure 2, the kriging model is established by using simulated data, and model parameters are obtained by the nonlinear optimization method. Thereafter, the original kriging model is further calibrated and validated. In the kriging surrogate model, the number of parameter θ is 6, the same as the dimension of the independent variable x. There are *p* = 7 regression coefficients, $\beta ={\left[\beta_{1},\beta_{2},\ldots ,\beta_{p}\right]}^{T}$. Table 1 shows the obtained 13 parameters of the kriging model before and after calibration. The output parameters of the original kriging model, Krig_θ and Krig_β, are used as the initial values of the calibrated model parameters. The parameters Calib_θ and Calib_β of the kriging calibration model are then obtained through calibration with a set of new data. Moreover, the variance values of the original kriging model and calibration model are calculated as $\sigma_{z\_krig}^{2}=1.125\times 10^{13}$ and $\sigma_{z\_calib}^{2}=2.150\times 10^{12}$, respectively.

### 3.2. Analysis of Temperature Prediction

**Figure 4:**
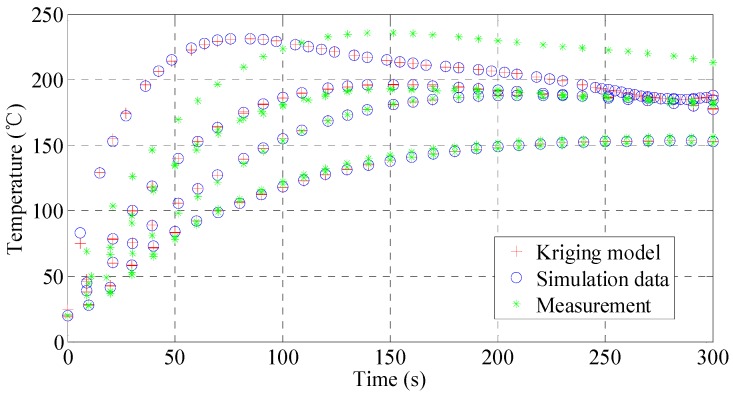
Sample data and kriging model prediction.

The response values of sample points and test points are predicted by the kriging model. Figure 4 shows the comparison of the predicted values, the numerical simulation and the experimental data. It is observed that the predicted results are consistent with the trend of the simulation and experimental data, but there is a significant difference between the prediction and experimental data in some sample points. Furthermore, the average error between the predicted values and the simulation data is less than 1 °C and its variance is less than 1. In particular, the model prediction agrees very well with the simulated data after 100 s, with the temperature error of less than 1 °C and its relative errors below 1%. However, due to the data uncertainty in the beginning, the model shows a significant prediction error before 30 s, up to 7.8 °C. This implies that model calibration using the experimental data is needed.

**Figure 5:**
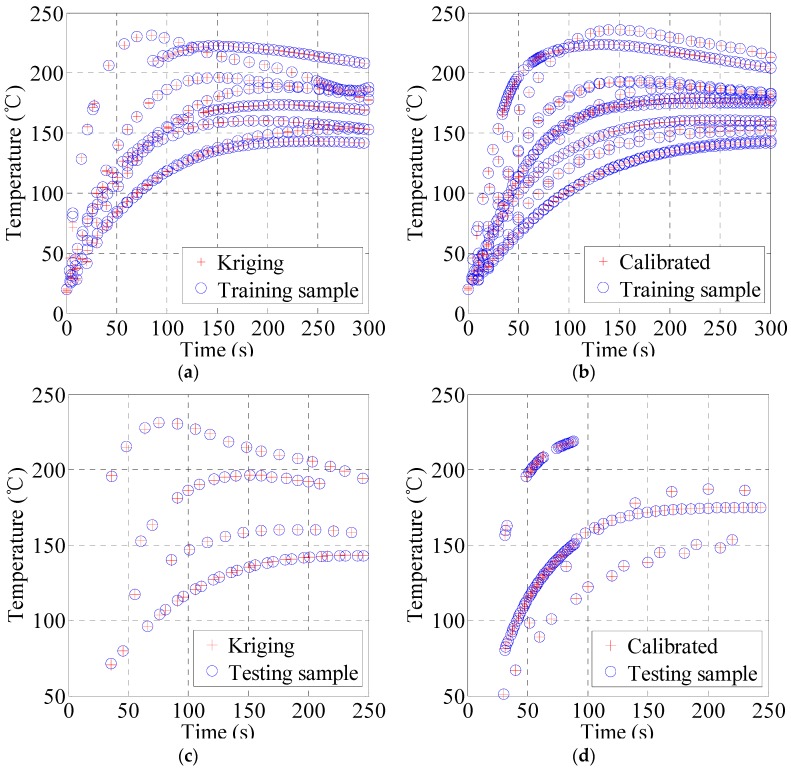
Sample data and kriging model prediction. (**a**) kriging prediction and training sample data; (**b**) calibrated kriging prediction and training sample data; (**c**) kriging prediction and test sample data; (**d**) calibrated kriging prediction and test sample data.

In order to investigate the difference between the simulation and experimental data, a comparison study of prediction and measurement data is conducted. Figure 5 shows the temperature responses of the full sample points and the corresponding predicted values from both the initial and calibration kriging models. Figure 5a,b compare the predicted values of the two kriging models with the corresponding training samples. Similar to Figure 4, the prediction values of the two kriging models show better continuity and consistency with the sample data. However, from Figure 5a, it is clearly observed that the consistency of a few points data in the first 50 s is relatively low, and the maximum temperature error is 7.8 °C. Figure 5b is the predicted curve of the calibrated model. It is observed that the average temperature error and variance are less than 0.5. Also in comparison to the initial kriging model data shown in Figure 5a, the calibrated model produces less individual errors in the first 30 s with the maximum temperature error of 3.9 °C. This study demonstrates that the accuracy of the kriging surrogate model has been improved by calibration with experimental samples.

Figure 5c,d illustrate the predicted results of the kriging model with the new test sample points. Figure 5c shows the predicted results for 70 test samples. Its temperature average error and maximum error is 0.27 °C and 1.15 °C, respectively, and its maximum relative error is less than 1%. Figure 5d shows the predicted results of the calibrated kriging model for 118 test samples. Its temperature average error and maximum error is 0.028 °C and 1.02 °C, respectively, and its maximum relative error is less than 1%. Obviously, the accuracy of the kriging model can be improved through calibrating with new experimental samples.

### 3.3. Variance Analysis of Predicted Values

The variance of the model prediction partly results from the uncertainty of the corresponding sample points and the sample sparsity. The smaller variance indicates the less uncertainty, namely, the model prediction may be more accurate in this region.

**Figure 6:**
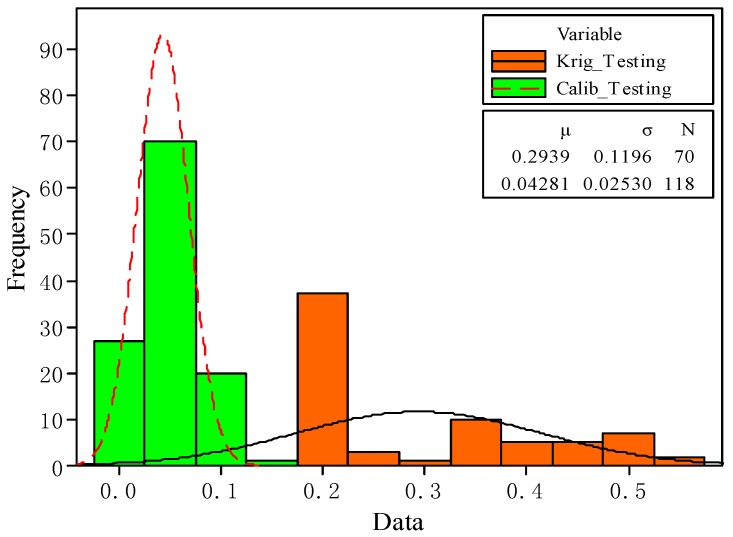
Variance test of kriging and calibrated models.

According to Equation (13), the variance values of model prediction at 70 simulated test samples and 118 experimental samples are obtained, as shown in Table 2. From the table, the mean and standard deviation of the variance values from the initial kriging model values are 0.2939 and 0.1196, respectively. It is observed that the mean and standard deviation values from the calibrated model prediction are much smaller, i.e., 0.0428 and 0.0253, respectively. In addition, the maximum variance of the calibration model is 0.1337, still less than the minimum variance 0.1798 obtained from the initial kriging model. Figure 6 shows the histogram of the two models’ variances, in the range of 0 and 0.6. It is indicated that all the variance values of the calibrated model are smaller than those from the initial kriging model. Therefore, the calibrated model provides more accurate prediction results than the initial kriging model.

### 3.4. Comparison of Friction Results

The comparison of the experimental results and simulation data in previous work [1] indicate that the friction plays a significant role in the injection process. Here, based on the predicted results of the kriging model, the influence of the friction on the temperature change of the injection mechanism is further investigated.

**Figure 7:**
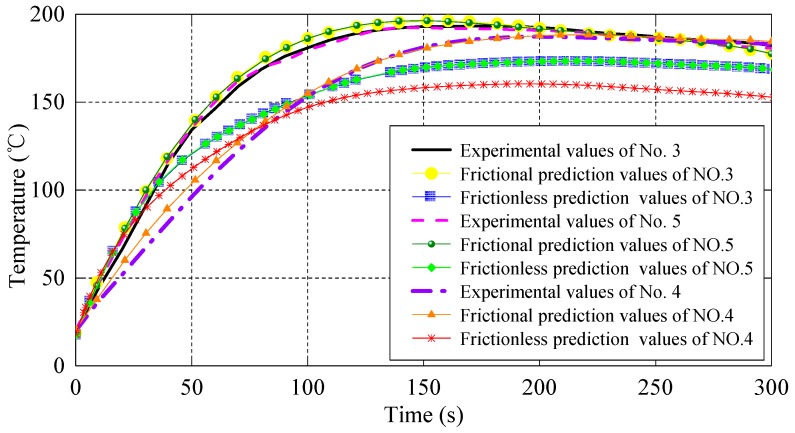
Temperature series in section A.

Figure 7 shows the temperature variation of the punch and the shot sleeve in section A predicted by the kriging surrogate model, where No. 3 and No. 5 represent the temperature variation of the shot sleeve, and No. 4 represents the temperature variation of the punch. Comparing the overall temperature curve of the punch and the shot sleeve, it is observed that the temperature of the shot sleeve is higher than that of the punch, and the former increases faster than the latter. This would be attributed to the different material properties and thermal conductivity of the two components. Under friction, the predicted values of the shot sleeve temperature are in good agreement with the experimental data. However, when ignoring friction, the maximum relative error is about 15%, which is much bigger than the value under friction, i.e., 5% at about 150 s. The similar observations are obtained from the punch temperature prediction. This study further indicates the influence of friction on the injection process.

**Figure 8:**
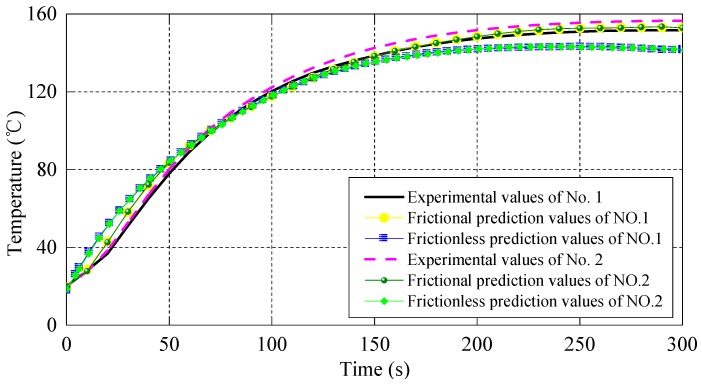
Temperature series of punch in section B.

Figure 8 shows the temperature curves of the punch in section B. It is observed that the temperature variation trend of all six curves is consistent. Considering friction, the predicted values are more in agreement with the experimental data than those without considering friction. Similarly, at the beginning of the injection process, there is a significant temperature error between the predicted and experimental data, and then the relative error decreases gradually with the value close to 1%.

## 4. Conclusions

According to the temperature spatiotemporal distributions of the injection mechanism in the injection process of squeeze casting, a MISO mathematical model is presented to investigate the uncertainty based on the kriging method. The model is employed to predict the temperature response values at different sample points. By comparing the predicted data with the sample data, the following conclusions are obtained:

1. The prediction results of the kriging surrogate model show that there are a few significant error values between the predicted and simulated data in the early injection stage, and, thereafter, the error decreases gradually. This phenomenon indicates the impact of the uncertainty on the temperature distribution of the injection mechanism in the injection process.
2. The variance mean and standard deviation obtained from the calibrated model are relatively smaller, which indicate that the calibration model is improved in terms of the prediction accuracy.
3. By a comparison study, the influence of friction on injection forming is further verified. By considering friction, the relative error between the model prediction and the experimental data at section A is obviously smaller than that obtained by ignoring friction.

In future research, the uncertainty quantification and its impact on the results will be investigated with real-world application scenarios.

## Figures and Tables

**Table 1:** Kriging model parameters obtained from simulation and calibration data.

Symbol	1	x1	x2	x3	x4	x5	x6
Independent Variable		x Axis	y Axis	z Axis	t	I_f_	Is
Krig_β (×10^3^)	8.416	0.341	−2.673	−1.325	−1.647	−7.556	0.557
Krig_θ	—	4.573	0.125	0.001	0.100	10.219	1.015
Calib_β (×10^3^)	−2.721	1.003	1.103	−0.198	0.485	2.562	−0.923
Calib_θ	—	7.476	0.0625	0.001	0.100	10.219	1.008

**Table 2:** Statistics of variance for kriging and calibrated models.

Model	Data Point	Mean Value	Standard Deviation	Minimum Value	Median	Maximum Value
Krig_Testing	70	0.2939	0.1196	0.1798	0.2116	0.5369
Calib_Testing	118	0.0428	0.0253	0.0134	0.0375	0.1337
